# Assessing post-abortion care in health facilities in Afghanistan: a cross-sectional study

**DOI:** 10.1186/s12884-015-0439-x

**Published:** 2015-02-03

**Authors:** Nasratullah Ansari, Partamin Zainullah, Young Mi Kim, Hannah Tappis, Adrienne Kols, Sheena Currie, Jaime Haver, Jos van Roosmalen, Jacqueline EW Broerse, Jelle Stekelenburg

**Affiliations:** Jhpiego/Afghanistan, Johns Hopkins University/Afghanistan, House 289, Street 3, Ansari Wat, Shar-e Naw, Kabul Afghanistan; Jhpiego/USA, Johns Hopkins University, Baltimore, USA; VU University Amsterdam, Amsterdam, Netherlands; Leeuwarden Medical Center, Department of Obstetrics & Gynecology, Leeuwarden, Netherlands

**Keywords:** Post-abortion care, Emergency obstetric and neonatal care, Afghanistan

## Abstract

**Background:**

Complications of abortion are one of the leading causes of maternal mortality worldwide, along with hemorrhage, sepsis, and hypertensive diseases of pregnancy. In Afghanistan little data exist on the capacity of the health system to provide post-abortion care (PAC). This paper presents findings from a national emergency obstetric and neonatal care needs assessment related to PAC, with the aim of providing insight into the current situation and recommendations for improvement of PAC services.

**Methods:**

A national Emergency Obstetric and Neonatal Care Needs Assessment was conducted from December 2009 through February 2010 at 78 of the 127 facilities designated to provide emergency obstetric and neonatal care services in Afghanistan. Research tools were adapted from the Averting Maternal Death and Disability Program Needs Assessment Toolkit and national midwifery education assessment tools. Descriptive statistics were used to summarize facility characteristics, and linear regression models were used to assess the factors associated with providers’ PAC knowledge and skills.

**Results:**

The average number of women receiving PAC in the past year in each facility was 244, with no significant difference across facility types. All facilities had at least one staff member who provided PAC services. Overall, 70% of providers reported having been trained in PAC and 68% felt confident in their ability to perform these services. On average, providers were able to identify 66% of the most common complications of unsafe or incomplete abortion and 57% of the steps to take in examining and managing women with these complications. Providers correctly demonstrated an average of 31% of the tasks required for PAC during a simulated procedure. Training was significantly associated with PAC knowledge and skills in multivariate regression models, but other provider and facility characteristics were not.

**Conclusions:**

While designated emergency obstetric facilities in Afghanistan generally have most supplies and equipment for PAC, the capacity of healthcare providers to deliver PAC is limited. Therefore, we strongly recommend training all skilled birth attendants in PAC services. In addition, a PAC training package should be integrated into pre-service medical education.

**Electronic supplementary material:**

The online version of this article (doi:10.1186/s12884-015-0439-x) contains supplementary material, which is available to authorized users.

## Background

Globally, 292,982 maternal deaths occurred in 2013, and 43,684 of these women lost their lives as a result of complications from abortion. Abortion remains among the leading causes of maternal death worldwide [1]. In Afghanistan, one of the 16 countries with the highest maternal mortality ratios in the world and the highest in south Asia, there is little data on the incidence of complications of spontaneous, incomplete, or unsafe abortion, In southeast Asia, the estimated abortion rate in 2008 was 36 per 1,000 women aged 15–44 years [2]. In Pakistan, which borders Afghanistan, analyses using indirect estimation techniques suggest an abortion rate of at least 29 per 1,000 women aged 15–49, and a national survey in 2006–07 revealed that 6% of maternal mortality could be attributed to abortion-related complications [3,4]. Although the 2010 Afghanistan Mortality Study did not report any maternal deaths directly caused by complications of abortion, it is likely that some of the 56% of maternal deaths due to hemorrhage and 5% of maternal deaths due to sepsis resulted from incomplete or unsafe abortions [5]. Other deaths from complications of abortion may not have been reported at all due to stigma associated with abortions and legal restrictions that only permit pregnancy termination to save the life of the mother [6,7].

Post abortion care (PAC) is an approach to reduce maternal mortality and morbidity from complications of spontaneous, incomplete, or unsafe abortion as part of the broader goal of improving women’s sexual and reproductive health and life [8]. The term ‘post abortion care’ was first used in 1991 in the context of integrating PAC with family planning services in order to break the cycle of unwanted pregnancies and improve the overall outcome of unsafe abortions. The essential components of PAC are: a) community and service provider partnerships for prevention of unwanted pregnancies and unsafe abortion; b) counseling to identify and address the emotional and physical health needs of women, c) treatment of incomplete abortion using manual vacuum aspiration (MVA) or misoprostol to remove retained products of conception; d) post-procedure family planning counseling and services, and e) links with other reproductive health care [8-10]. The Ministry of Public Health (MoPH) in Afghanistan recognizes MVA as an essential component, or signal function, of emergency obstetric and neonatal care (EmONC) and has sought to expand access to PAC as part of its efforts to improve the health of the Afghan people [11,12]. The national Reproductive Health Strategy includes PAC as a component of safe motherhood services, and national Intrapartum and EmONC Standards provide guidelines for skilled birth attendants (doctors and midwives) on the use of MVA for evacuation of retained products of conception up to 16 weeks [13-15].^a^

Despite the importance of PAC in averting complications and mortality and its promulgation in national policies, data on the provision and quality of PAC services at EmONC facilities in Afghanistan, including the technical capacity of providers, are scant. PAC is not reported in the Health Management Information System, and there is a lack of data on the incidence of abortion or provision of PAC in the country. The aim of this study is to assess the public health system’s readiness to provide PAC services, using data from a 2009–10 EmONC needs assessment. We hope that providing information on the availability of human resources, equipment and supplies, as well as the knowledge and skills of skilled birth attendants at designated EmONC facilities, will be of value to policy makers, health system managers and other stakeholders working to reduce preventable maternal mortality in Afghanistan.

## Methods

**Figure 1:**
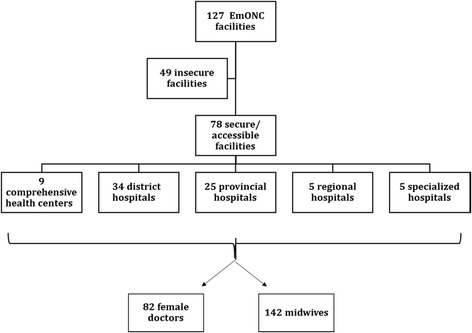
**Facilities and health workers included in the study sample.**

A cross-sectional national EmONC needs assessment was conducted in Afghanistan from December 2009 to February 2010 [16]. At that time, the national Health Management Information System database listed a total of 127 facilities nationwide that were expected to provide EmONC services, including removal of retained products of conception using MVA. These facilities included district, provincial, regional, and specialized national hospitals, as well as certain comprehensive health centers which were upgraded to provide comprehensive EmONC services in remote areas without a district hospital. Access to 49 facilities was limited because of security concerns; consequently, 78 of the 127 facilities were assessed. These included 9 comprehensive health centers with catchment populations of 30,000-60,000 people, 34 district hospitals with catchment populations of 100,000-300,000, 25 provincial hospitals, 5 regional hospitals and 5 specialized maternity hospitals located in the capital city. At each provincial, regional and specialized hospital, two doctors and two midwives responsible for providing EmONC services were randomly selected to participate in the study. At each district hospital and comprehensive health center, two providers were selected, including a doctor if available. No doctor was available at the time of data collection at 13 of the district hospitals and 3 of the comprehensive health centers, resulting in a total sample size of 82 doctors and 142 midwives—all female—at 78 facilities (Figure 1).

The assessment team consisted of 6 female doctors and 38 female midwives. All data collectors had at least five years of experience in their respective professions and with various international and national organizations, as well as prior training on and experience with research and data collection. The data collectors were trained for one week in data collection tools and techniques including research ethics.

The assessment used modified tools based on the Averting Maternal Death and Disability (AMDD) Program Needs Assessment Toolkit developed by Columbia University to document the availability of equipment, drugs, supplies and human resources for all EmONC services, including PAC [17]. The tools were approved in a workshop with MoPH officials and reproductive health stakeholders and pilot-tested during the training for data collectors.

To investigate providers’ proficiency in provision of high-quality PAC, assessors first interviewed all selected providers with three open-ended questions to identify the most common complications of unsafe or incomplete abortion (4 items), steps to take in examining and managing women with complications of unsafe or incomplete abortion (9 items), and information that should be provided to all women treated for incomplete or unsafe abortions (7 items) (Additional file 1: Provider Interview Guide). A total knowledge score was calculated for each provider as the percentage of all 20 items offered, without prompting, in response to the questions. Assessors then observed the providers perform MVA on an anatomical model and recorded their skills on a nine-part observation checklist (Additional file 2: Observation Checklist). Each part documented whether the provider completed a set of required tasks or not (ranging from as few as two tasks or as many as 19 tasks, depending on the complexity of the step); missing a task was considered failure to correctly complete a crucial step of PAC. An overall skills score was calculated for each provider as the percentage of 9 steps completed correctly. Because MVA is the PAC procedure promoted in national policies, proficiency in other procedures for treatment of incomplete abortion, specifically removal of retained products of conception through dilation and curettage or treatment with misoprostol, were not assessed.

Descriptive statistics were used to summarize facility characteristics, including the availability of trained staff and resources for high quality PAC provision. Student *t*-test and chi-square tests were used to identify differences in resource availability, provider knowledge, and provider skills across facility types. Finally, bivariate and multivariate linear regression models were used to assess the relationship between providers’ PAC knowledge and skills and characteristics of the health facility (type of facility and PAC caseload) and the provider (cadre, years of experience providing EmONC, training on MVA, and confidence in performing the procedure). The analyses were conducted using STATA 11.

The study protocol was approved by the institutional review boards of the Afghanistan Public Health Institute (#112595) and the Johns Hopkins School of Public Health (#2333).

## Results

### Caseload, supplies, equipment and staffing

The number of women who received PAC in the previous year at each EmONC facility varied widely, from 0 to 1,457, with a median of 87. No statistically significant differences in PAC caseload were observed across the five facility types (Table 1). Availability of supplies required for PAC varied. Overall, 78% of facilities had manual vacuum aspirators, and 75% had flexible cannulas; however, only 70% of facilities had both items necessary for PAC provision. Uterotonics required to treat hemorrhage after abortion were available at 88% of facilities, essential antibiotics were available at 97% of facilities, paracetamol for post-procedure pain management was available at 91% of facilities, and both short-acting and long-acting forms of modern contraceptives were available at 94% of facilities. Availability of both short- and long-acting contraceptives varied significantly across facility types, with the greatest gaps in supply documented at regional hospitals.

**Table 1:** **Facility characteristics: caseloads and percentage of facilities with supplies and equipment available, by facility type**

**Characteristics**	**Total (n = 78)**	**Comprehensive health centers (n = 9)**	**District hospitals (n = 34)**	**Provincial hospitals (n = 25)**	**Regional hospitals (n = 5)**	**Specialized hospitals (n = 5)**	**p-value**
**No. of deliveries in last 12 months**							
Range (median)	38–20,531 (1145)	129-468 (295)	38-3,501 (714.5)	230 – 10,943 (2,132)	4,005 – 15,202 (7,047)	1,592 – 20,531 (10,643)	
Mean (SD)	2,547 (434)	307 (40)	1,061 (155)	2,298 (420)	8,243 (1,911)	11,738 (3,324)	p<0.001
**No. of PAC cases in last 12 months**							
Range (median)	0-1,457 (87)	0-1,109 (30)	0-412 (84)	0-1,037 (98)	496-1,439 (734)	0-1,457 (214)	
Mean (SD)	224 (38)	148 (120)	110 (19)	204 (50)	799 (171)	656 (325)	0.084
**Provided PAC services in last 12 months**	84.6	88.9	82.3	84.0	100.0	80.0	0.868
**Supplies and equipment available**							
Uterotonic	92.0	88.9	91.2	100.0	50.0^e^	100.0	0.016
*Ergometrine*	88.0	88.9	85.3	95.7	50.0^e^	100.0	0.104
*Oxytocin*	63.0	88.9	82.4	91.3	50.0^e^	80.0	0.326
Paracetemol	90.8	88.9	85.3	95.8 ^c^	100.0	80.0	0.573
IV fluids	98.7	100.0	97.1	100.0	100.0	100.0	0.860
*Syringes (1, 2, 5, 10mmL)*	98.9	100.0	94.1	92.0	100.0	100.0	0.828
Catheter for IV line	88.5	77.8	82.4	96.0	100.0	100.0	0.293
Vacuum aspirators	76.6	77.8	67.7	86.7^d^	100.0	80.0	0.449
O-ring lubricant (silicone)	58.7	50.0^a^	48.5^b^	66.7^c^	100.0	60.0	0.213
O-ring lubricant (other)	62.7	75.0^a^	45.5	75.0^c^	80.0	80.0	0.110
Flexible cannulae, *(2 sizes)*	75.0	87.5^a^	66.7^b^	76.0^c^	80.0	80.0	0.754
Aspirators and cannulae	70.1	77.8	64.7	70.8	80.0	80.0	0.878
Essential antibiotics	97.4	100.0	97.1	96.0	100.0	100.0	0.948
At least one form of modern contraceptives	94.7	100.0	97.1	96.0	60.0	100.0	0.025
*Short acting contraceptives*	93.6	100.0	97.1	92.0	60.0	100.0	p<0.001
*Long acting reversible contraceptives*	93.6	100.0	97.1	96.0	40.0	100.0	p<0.001

^a^n = 8; ^b^n = 33; ^c^n = 24; ^d^n = 23; ^e^n = 4.

At the time of the survey, all facilities reported having at least one staff person that provides PAC. Midwives were the cadre most consistently reported to perform this service across facility types, followed by general doctors. Only 11% of comprehensive health centers and 17.6% of district hospitals had obstetrician/gynecologists that performed MVA (Table 2). All hospitals had at least two midwives; however, only 80% of midwives at specialized hospitals, 76% of midwives at provincial hospitals and 85% of midwives at district hospitals provided MVA. All comprehensive health centers had at least one midwife that provided MVA. Overall, 71% of providers reported having been trained in PAC, and 68% of all providers felt confident in their ability to perform these services (Table 3).

**Table 2:** **Availability of providers to perform PAC: Percent distribution of facilities by number of staff employed and by number who provide MVA, according to cadre and facility type**

**Cadre**	**# of staff**	**Comprehensive health centers (n = 9)**		**District hospitals (n = 34)**		**Provincial hospitals (n = 25)**		**Regional hospitals (n = 5)**		**Specialized hospitals (n = 5)**	
		**Employed**	**Provide MVA**	**Employed**	**Provide MVA**	**Employed**	**Provide MVA**	**Employed**	**Provide MVA**	**Employed**	**Provide MVA**
**General doctor**	0	---	44.4	8.8	35.3	4.0	52.0	---	20.0	20.0	20.0
	1	22.2	---	2.9	---	---	---	---	---	---	---
	2+	77.8	55.6	88.2	64.7	96.0	48.0	100.0	80.0	80.0	80.0
**Obstetrician/ gynecologist**	0	88.9	88.9	73.5	82.4	32.0	48.0	---	---	---	20.0
	1	---	---	20.6	14.7	40.0	28.0	---	---	---	---
	2+	11.1	11.1	5.9	2.9	28.0	24.0	100.0	100.0	100.0	80.0
**Surgeon**	0	66.7	88.9	20.6	88.2	8.0	96.0	20.0	100.0	---	100.0
	1	33.3	11.1	61.8	11.8	20.0	4.0	---	---	20.0	---
	2+	---	---	17.6	---	72.0	---	80.0	---	80.0	---
**Nurse**	0	0.0	88.9	11.8	94.1	4.0	96.0	20.0	100.0	---	100.0
	1	33.3	---	---	---	8.0	4.0	---	---	---	---
	2+	66.7	11.1	88.2	5.9	88.0	---	80.0	---	100.0	---
**Midwife**	0	---	---	---	14.7	---	24.0	---	---	---	20.0
	1	11.1	11.1	---	---	---	---	---	---	---	---
	2+	88.9	88.9	100.0	85.3	100.0	76.0	100.0	100.0	100.0	80.0

**Table 3:** **Percent of providers who report having received training in PAC and who feel confident in providing PAC**

	**Comprehensive health centers (n = 13)**	**District hospitals (n = 57)**	**Provincial hospitals (n = 73)**	**Regional hospitals (n = 17)**	**Specialized hospitals (n = 17)**	**Total (n = 177)**	**p-value**
**Trained in PAC**	70.6	61.8	72.3	95.0	66.7	70.5	0.073
**Confident in providing PAC**	64.3	68.4	54.8	87.5	64.7	62.9	0.111

### Provider knowledge

Providers were able to identify an average of 66% of the most common complications of unsafe or incomplete abortion (i.e., sepsis, bleeding, genital injuries, and shock) and named 57% of the steps needed for examining and treating a woman with these complications. (See Table 4 for the list of items.) Providers correctly described less than half (46%) of the information on birth spacing, family planning, and consequences of unsafe abortion that should be provided to PAC patients. Knowledge of what steps to take in examining and managing women with post-abortion complications varied significantly across facility types, as did the total knowledge score. On average, providers at provincial and specialized hospitals demonstrated greater knowledge of tasks required for high quality PAC than staff at other facility types.

**Table 4:** **PAC knowledge scores: Mean percent of items known by providers, by facility type**

**PAC knowledge**	**Mean score (SD)**	**Comprehensive health centers (n = 13)**	**District hospitals (n = 57)**	**Provincial hospitals (n = 73)**	**Regional hospitals (n = 17)**	**Specialized hospitals (n = 17)**	**p-value**
**Can identify immediate complications of unsafe or incomplete abortion:** sepsis, bleeding, genital injuries, shock (percent of 4 items correct)	65.5 (1.6)	60.3	60.7	67.8	63.8	77.5	0.058
**Can identify steps to take in examining and treating a woman with complications from unsafe or incomplete abortion**: assess vaginal bleeding and vital signs, begin IV fluids and antibiotics, do manual vacuum aspiration and conventional evacuation with curettage, provide counseling and refer (percent of 9 items correct)	57.4 (1.5)	50.3	49.7	63.2	55.6	63.9	0.001
**Knows what information to provide patients who were treated for an incomplete or unsafe abortion:** Information on birth spacing, family planning, consequences of unsafe abortion, and social support (percent of 7 items correct)	46.1 (1.7)	40.3	42.2	49.0	49.3	47.1	0.36
**Total knowledge score** (percent of 20 items correct)	*55.1 (1.3)*	*48.8*	*49.3*	*59.2*	*55.0*	*60.8*	*0.007*

### Provider skills

Less than one-third (31%) of providers correctly demonstrated the tasks required for high quality PAC during the simulated procedure (Table 5). Of all of the stages of PAC evaluated, providers scored the lowest on the MVA procedure steps; only 13% of providers adequately demonstrated the tasks required for safe and effective MVA. Thirty-six percent of providers correctly demonstrated pre-procedure and post-procedure infection prevention tasks, and 43% of providers correctly demonstrated initial assessment skills, including introducing themselves to the patient, assessing her for signs of shock and other life-threatening conditions, and stabilizing her if any complications are identified. Only 25% of providers correctly demonstrated medical evaluation skills including taking a reproductive health history, performing physical examination and indicated laboratory tests, and providing the woman with information about her condition and what to expect from PAC procedures. There were no significant differences in provider skills across facility types.

**Table 5:** **Percent of providers who correctly demonstrate skills during simulation of PAC on an anatomical model, by facility type**

**PAC skills area (number of tasks)**	**All providers (n = 177)**	**Comprehensive health center (n = 13)**	**District hospital (n = 57)**	**Provincial hospital (n = 73)**	**Regional hospital (n = 17)**	**Specialized hospital (n = 17)**	**p-value**
**Initial assessment** (3 tasks)	42.4	46.2	36.8	53.4	29.4	23.5	0.089
**Medical evaluation** (6 tasks)	25.4	7.7	26.3	28.8	23.5	23.5	0.614
**Preparation for procedure** (10 tasks)	44.1	53.9	42.1	42.5	47.1	47.1	0.939
**Pre-procedure infection prevention** (10 tasks)	36.2	30.8	28.1	41.1	41.2	41.2	0.571
**Administering paracervical block (if needed)** (8 tasks)	23.7	15.4	22.8	24.7	41.2	11.8	0.312
**MVA procedure** (19 tasks)	13.1	15.4	10.5	12.3	17.7	17.7	0.902
**MVA post-procedure infection prevention** (9 tasks)	36.2	61.5	28.1	37.0	47.1	29.4	0.166
**MVA post-procedure monitoring** (2 tasks)	33.9	30.8	24.6	46.6	23.5	23.5	0.058
**MVA post-procedure counseling** (4 tasks)	23.2	30.8	26.3	23.3	23.5	5.9	0.464
**Total skill score** (percent of skill areas completed correctly)	*30.9*	*32.5*	*27.3*	*34.4*	*32.7*	*24.8*	*0.975*

### Variables associated with PAC knowledge and skills

Modeling the relationship between provider and facility-level characteristics and PAC knowledge and skills revealed small, but statistically significant associations between provider characteristics and PAC capacity. Bivariate analyses suggest that cadre is significantly associated with provider knowledge, while training in PAC is significantly associated with both provider knowledge and skills (Table 6). When provider and facility-level characteristics are considered together in multivariate linear regression models, only training is significantly associated with PAC knowledge (p = 0.016) and skills (p = 0.011).

**Table 6:** **Bivariate and multivariate associations between provider and facility characteristics and provider knowledge and clinical skills in PAC**

**Characteristics**	**Bivariate analysis**						**Multivariate analysis**					
	**Overall knowledge**			**Overall clinical skills**			**Overall knowledge**			**Overall clinical skills**		
	**Coef**	**SE**	***P***	**Coef**	**SE**	***P***	**Coef**	**SE**	***P***	**Coef**	**SE**	***P***
**Provider cadre** (ref: midwife)												
Doctor	0.061	0.027	0.029	−0.018	0.042	0.670	0.044	0.029	0.135	−0.041	0.046	0.380
**Number of years of experience as EmONC provider**	−0.002	0.002	0.282	−0.002	−0.640	0.526	−0.001	0.002	0.654	0.000	0.003	0.911
**Provider trained in MVA**	0.083	0.029	0.004	0.157	0.049	0.002	0.087	0.036	0.016	0.154	0.060	0.011
**Health facility type** (ref: Comprehensive Health Center)												
District Hospital	0.005	0.053	0.930	−0.052	0.083	0.534	0.002	0.060	0.978	0.025	0.094	0.792
Provincial Hospital	0.104	0.051	0.045	0.019	0.082	0.814	0.082	0.057	0.151	0.103	0.090	0.255
Regional Hospital	0.062	0.064	0.339	0.002	0.100	0.984	0.063	0.077	0.412	0.107	0.115	0.352
Specialized Hospital	0.119	0.064	0.066	−0.076	0.100	0.445	0.083	0.074	0.264	0.024	0.115	0.833
**Annual number of PAC cases reported at facility**	0.000	0.000	0.076	0.000	0.000	0.555	0.000	0.000	0.508	0.000	0.000	0.726

## Discussion

Ensuring the availability of resources and knowledgeable, skilled health professionals to provide high quality PAC is essential to reduce mortality and morbidity due to complications of unsafe or incomplete abortions. This study shows that while most designated EmONC facilities in Afghanistan have the supplies and equipment needed for PAC, the capacity of healthcare providers to deliver this service is limited. At each visited facility, at least one health care provider was available to perform PAC services. However, the knowledge and skills of most providers were insufficient to deliver quality PAC. The gaps documented in providers’ skills are consistent with the results of a 2008 pre-service midwifery evaluation in Afghanistan, which found that midwives graduating from the Institute of Health Sciences had limited capacity to provide MVA [18]. A study of the quality of PAC services in Ethiopia reported findings similar to this study. Although facilities had appropriate medical equipment and supplies for PAC, the training of most service providers fell short: the training did not follow current PAC guidance and focused on the general management of PAC clients without providing important information on danger signs, follow-up needs, and care associated with pain management. However, this study did show better results than previous studies in Ethiopia, indicating that PAC can be improved if dedicated efforts are made by the MoPH and health development partners [19].

Training was significantly associated with providers’ PAC knowledge and skills in multivariate analyses. This suggests that current training strategies are having a positive impact and, even more importantly, that strengthened training has the potential to raise low knowledge and skills levels. Although PAC is an integral part of the existing EmONC in-service training package in Afghanistan, in-service training is not mandatory and only one day of this 21-day refresher course is allocated to PAC management. During this one day, participants learn pre-procedure and post-procedure tasks and complete a clinical practicum [20]. Low skill levels observed in this assessment may indicate that a single day is not sufficient for health workers to develop skills required for MVA. Coverage of PAC in pre-service medical and midwifery education programs in Afghanistan is even more limited. While pre-service midwifery education does include PAC training, a qualitative study found that midwifery students had limited opportunity to practice some skills, and half the respondents felt unprepared in the management of certain EmONC skills [21]. Pre-service medical education does not cover PAC, and the only instruction on PAC services that doctors receive is during in-service training. A separate analysis of the EmONC assessment data used in this study suggests that doctors and midwives are equally unprepared to provide PAC; there were no significant differences in the performance scores on a range of EmONC topics including MVA [22].

To address the poor performance of providers, more and better training is needed. First and foremost, national policies should be updated in line with international standards, and training packages revised accordingly. In addition to incorporating recommendations for treatment of retained products of conception with misoprostol, policies should remove recommendations for use of paracetamol as a post-procedure pain reliever, as new evidence shows this is ineffective [15]. Furthermore, low knowledge of what information should be provided to PAC clients on birth spacing and family planning also suggests that counseling and provision of contraceptives may not be widely recognized as an essential component of high-quality PAC. Research in other settings has shown that improving contraceptive counseling training for skilled birth attendants and increasing access to multiple contraceptive methods on site may reduce women’s risk of unwanted pregnancy and, in turn, abortion [23,24].

The Afghanistan MoPH should collaborate with the Ministry of Higher Education to add a PAC training package to the pre-service medical education curriculum. In addition, the MoPH should revise the EmONC in-service training package or consider making PAC a separate training module so that non-surgical components of PAC, such as counseling and respectful maternity care, infection prevention, and family planning, can be more fully addressed. Developing a more extensive PAC training package that includes medical treatment of incomplete abortion with sublingual or oral misoprostol could also help to improve the availability and quality of PAC services, as this treatment costs less and is easier to perform than other options [25-27]. A study in Ghana supports the idea of offering a separate training module on PAC; it found that training midwives in Emergency Obstetric Care did not effectively impart PAC skills and that focused PAC/MVA training appeared to be more likely than safe motherhood or lifesaving skills training to lead to delivery of PAC services [19]. Similarly, an evaluation in Nepal found that nurses who completed a 14-day in-service training on PAC provided high quality MVA procedures with confidence [28]. Longer and more in-depth training should be extended to the workplace, via supportive supervision and post-training follow-up, in order to ensure provider competency needed to provide high quality PAC services. Repetition and reinforcement of training is essential, because recent studies show that repetitive, time-spaced training reinforces essential messages, provides more opportunities to practice skills, and creates mechanisms for fostering interactions, and that it is more effective than single-event training [29].

Finally, to strengthen the health system’s capacity to provide high quality PAC and ensure access to all women who need the service, additional information is needed on the incidence of abortion, PAC service utilization, and barriers to care seeking in Afghanistan. To gather these data, PAC service information should be integrated into the Health Management Information System and also into the National Monitoring Checklist, a monitoring tool that measures the quality of health services in Afghanistan. Universal coverage of lifesaving interventions needs to match with comprehensive emergency care and improvement of quality of maternal health care if the goal is to substantially reduce maternal mortality [30].

This study provides important information on the current capacity of the public health system in Afghanistan to provide PAC. However, interpretation of the results is subject to certain limitations. First, although the study was designed to be national in scope, security concerns limited the number and locations of facilities assessed. Second, a clinical simulation with anatomical models may not reflect providers' actual performance on the job, especially how well they counsel and interact with patients, which is a critical part of PAC. Finally, the sampling strategy—which selected similar numbers of providers from each facility—may underrepresent providers who work at larger, busier facilities and may have stronger skills. Despite these limitations, this study fills a gap in research on the current state of PAC in Afghanistan and offers recommendations for improvement of services to save women's lives.

## Conclusions

While designated EmONC facilities in Afghanistan generally have most of the supplies and equipment needed for PAC, this study shows that the capacity of healthcare providers to deliver PAC is limited. Recommendations include training of all midwives and doctors in PAC through integration of PAC training packages in pre-service medical education and expansion of PAC training components in pre-service midwifery education, as well as enhanced in-service training for both cadres.

## Endnote

^a^ It should be noted that this guideline is not consistent with current WHO guidelines, which state that if uterine size at the time of treatment is equivalent to a pregnancy of gestational age 13 weeks or less, either vacuum aspiration or treatment with misoprostol is recommended for women with incomplete abortion [15].

## Additional files

Additional file 1:
**Provider Interview Guide.**
Additional file 2:
**Observation Checklist.**
