# Supplementary material for: Assessing post-abortion care in health facilities in Afghanistan: a cross-sectional study
Source: BMC Pregnancy Childbirth. 2015 Feb 3;15:6. doi: 10.1186/s12884-015-0439-x (PMC4320442; doi:10.1186/s12884-015-0439-x)
Supplement: Additional file 1: — Provider Interview Guide. [file 12884_2015_439_MOESM1_ESM.pdf]

innovating to save lives
Jhpiego
an affiliate of Johns Hopkins University

PROVIDER INTERVIEW GUIDE*

* Excerpted from Afghanistan Emergency Obstetric and Newborn Care Needs Assessment Provider Interview Guide

<u>Interview Data</u>

1. Assessor ID: ____________________ 2.Date: _____________________
3. Start Time: ____________________ 4. End Time: _____________________

<u>Health Facility Data</u>

5. Health Facility National ID Number ____________________ 6. Health Facility Type: CHC, DH, PH, RH, SH
7. Province: ____________________ 8. District: ____________________

<u>Provider Data</u>

9. Study-Assigned Provider ID Number ____________________ 10. Cadre: (Circle one) Doctor, Midwife, Other
11. Time provider has worked at this health facility __ __ years __ __ months 12. Number of years and months in clinical EmONC practice since qualification __ __ years __ __ months

INSTRUCTIONS TO THE ASSESSOR/INTERVIEWER:

- *Ask the questions. Do <u>not</u> read aloud the answers unless the question specifies that the answers be read aloud.*
- *Circle the correct number corresponding to the response given in each cell.*
- *If NA is circled, please explain why in the Comments section at the end of this checklist.*

No.	Question	Response		
	What are the immediate complications of an unsafe abortion?<br>*(Circle all spontaneous answers and ask: "Anything else?")*	a. Sepsis<br>b. Bleeding<br>c. Genital injuries<br>d. Shock	Mentioned<br>1<br>1<br>1<br>1	Did not Mention<br>0<br>0<br>0<br>0
	When you see a woman with complications from an unsafe or incomplete abortion, what do you do?<br>*(Circle all spontaneous answers and ask: Anything else?)*	a. Do a vaginal exam<br>b. Assess vaginal bleeding<br>c. Assess vital signs<br>d. Begin IV fluids<br>e. Begin antibiotics<br>f. Do manual vacuum aspiration (MVA)<br>g. Do conventional evacuation with curettage<br>h. Provide counseling<br>i. Refer	Mentioned<br>1<br>1<br>1<br>1<br>1<br>1<br>1<br>1<br>1	Did not Mention<br>0<br>0<br>0<br>0<br>0<br>0<br>0<br>0<br>0
	What information do you give patients who were treated for an incomplete or unsafe abortion?<br>*(Circle all spontaneous answers and ask: Anything else?)*	a. Information on how to prevent RTI/HIV<br>b. Information about when a woman can conceive again<br>c. Counseling on FP and services<br>d. Refer for FP to receive contraceptive method if the woman desires this<br>e. Info. On waiting at least 6 months before becoming pregnant again<br>f. Information on social support<br>g. Information about the consequences of an unsafe abortion	Mentioned<br>1<br>1<br>1<br>1<br>1<br>1<br>1	Did not Mention<br>0<br>0<br>0<br>0<br>0<br>0<br>0
