# Supplementary material for: Assessing post-abortion care in health facilities in Afghanistan: a cross-sectional study
Source: BMC Pregnancy Childbirth. 2015 Feb 3;15:6. doi: 10.1186/s12884-015-0439-x (PMC4320442; doi:10.1186/s12884-015-0439-x)
Supplement: Additional file 2: — Observation Checklist. [file 12884_2015_439_MOESM2_ESM.pdf]

innovating to save lives

Jhpiego

an affiliate of Johns Hopkins University

OBSERVATION CHECKLIST: MANUAL VACUUM ASPIRATION FOR POST ABORTION CARE*

Observation Data:

Assessor/Observer ID Number:	
0a. Health Facility Code:	
0b. Health Facility Type: Circle one:	CHC, DH, PH, RH, SH
0c. Province:	
0d. District:	
0e. Date in Afghan Calendar:	
0f. Cadre of Provider Being Observed: (Midwife, Doctor, etc.) (Write in	
0g. Number of months or years provider has worked at health facility	

INSTRUCTIONS TO THE ASSESSOR/OBSERVER:

- *Please circle the correct number in each cell.*
- *Circle 1 or Yes if step/task is performed satisfactorily according to the standard procedure or guidelines.*
- *Circle 0 or No if step/task is not performed satisfactorily according to the standard procedure or guidelines OR if step/task is not performed at all/not observed.*
- *If NA or circled, please explain why in the Comments section at the end of this checklist.*

Note: The provider may perform some of the steps simultaneously, as when explaining to the woman what is being done while it is being done.

STEP/TASK	YES	NO	NA
**I. INITIAL ASSESSMENT**			
i. Greet the woman respectfully and with kindness and introduce yourself	1	0	9
ii. Assess patient for shock and other life-threatening conditions.	1	0	9
iii. If any complications are identified, stabilize patient and transfer, if necessary.	1	0	9
**II. MEDICAL EVALUATION**			
i. Take a reproductive health history.	1	0	9
ii. Perform limited physical (heart, lungs and abdomen) and pelvic examinations.	1	0	9
iii. Perform indicated laboratory tests.	1	0	9
iv. Give the woman information about her condition and what to expect.	1	0	9
v. Discuss her reproductive goals, as appropriate.	1	0	9
vi. If she is considering an IUD (Circle 9 if she is not considering IUD):<br>She should be fully counseled regarding IUD use.<br>The decision to insert the IUD following the MVA procedure will be dependent on the clinical situation.	1	0	9
**III. PREPARATION FOR PROCEDURE**			
i. Tell the woman (and her support person) what is going to be done, listen to her and respond attentively to her questions and concerns.	1	0	9
ii. Provide continual emotional support and reassurance, as feasible.	1	0	9
iii. Tell her she may feel discomfort during some of the steps of the procedure and you will tell her in advance.	1	0	9

iv. Give an Paracetamole 500 mg (Analgesic) to the woman 30 minutes before the procedure.	1	0	9
v. Pre-medication has been given to the patient	1	0	9
vi. Ask about allergies to antiseptics and anesthetics.	1	0	
vii. Determine that required sterile or high-level disinfected instruments are present.	1	0	9
viii. Make sure that the appropriate size cannula and adapters are available.	1	0	9
ix. Check the MVA syringe and charge it (establish vacuum).	1	0	9
x. Check that patient has recently emptied her bladder.	1	0	9
IV. **PRE-PROCEDURE INFECTION PREVENTION**			
i. Check that patient has thoroughly washed and rinsed her perineal area.	1	0	9
ii. Put on personal protective barriers.	1	0	9
iii. Wash hands thoroughly with soap and water and dry with a clean, dry cloth or air dry.	1	0	9
iv. Put high-level disinfected or sterile surgical gloves on both hands.	1	0	9
v. Arrange sterile or high-level disinfected instruments on sterile tray or in high-level disinfected container.	1	0	9
vi. Give oxytocin 10 units IM; ergometrine 0.2 mg IM (check WHO)	1	0	9
vii. Perform bimanual pelvic examination, checking the size and position of uterus and degree of cervical dilation.	1	0	9
viii. Insert the speculum and remove blood or tissue from vagina using sponge forceps and gauze.	1	0	9
ix. Apply antiseptic solution to cervix and vagina two times using gauze or cotton sponge.	1	0	9
x. Remove any products of conception (POC) from the cervical os and check cervix for tears.	1	0	9
V. **ADMINISTERING PARACERVICAL BLOCK (WHEN NECESSARY)**			
i. Prepare 20 mL 0.5% lignocaine solution without adrenaline.	1	0	9
ii. Draw 10 mL of 0.5% lignocaine solution into a syringe.	1	0	9
iii. If using a single-toothed tenaculum, inject 1 mL of lignocaine solution into the anterior or posterior lip of the cervix (the 10 o'clock or 12 o'clock position is usually used).	1	0	9
iv. Gently grasp anterior lip of the cervix with a single-toothed tenaculum or vulsellum forceps (preferably, use ring or sponge forceps if incomplete abortion).	1	0	9
v. With tenaculum or vulsellum forceps on the cervix, use slight traction and movement to help identify the area between the smooth cervical epithelium and the vaginal tissue.	1	0	9
vi. Insert the needle just under the epithelium and aspirate by drawing the plunger back slightly to make sure the needle is **not** penetrating a blood vessel.	1	0	9

vii. Inject about 2 ml of a 0.5% lignocaine solution just under the epithelium, not deeper than 3 mm, at 3, 5, 7 and 9 o'clock.	1	0	9
viii. Wait 2 minutes and then pinch the cervix with the forceps. (If the woman feels the pinch, wait 2 more minutes and then retest.)	1	0	9
**VI. MVA PROCEDURE**			
i. Inform woman of each step in the procedure prior to performing it.	1	0	9
ii. Gently apply traction on the cervix to straighten the cervical canal and uterine cavity.	1	0	9
iii. If necessary, dilate cervix using progressively larger cannula.	1	0	9
iv. While holding the cervix steady, push the selected cannula gently and slowly into the uterine cavity until it just touches the fundus (not more than 10 cm). Then withdraw the cannula slightly away from the fundus.	1	0	9
v. Attach the prepared syringe to the cannula by holding the cannula in one hand and the tenaculum and syringe in the other. Make sure cannula does not move forward as the syringe is attached.	1	0	9
vi. Release the pinch valve(s) on the syringe to transfer the vacuum through the cannula to the uterine cavity.	1	0	9
vii. Evacuate any remaining contents of the uterine cavity by rotating the cannula and syringe from 10 to 2 o'clock and moving the cannula gently and slowly back and forth within the uterus.	1	0	9
viii. If the syringe becomes half full before the procedure is complete, detach the cannula from the syringe. Remove **only** the syringe, leaving the cannula in place.	1	0	9
ix. Push the plunger to empty POC into the strainer.	1	0	9
x. Recharge syringe, attach to cannula and release pinch valve(s).	1	0	9
xi. Check for signs of completion (red or pink foam, no more tissue in cannula, a "gritty" sensation and uterus contracts around the cannula). Withdraw the cannula and MVA syringe gently.	1	0	9
xii. Remove cannula from the MVA syringe and push the plunger to empty POC into the strainer.	1	0	9
xiii. Remove tenaculum or forceps from the cervix before removing the speculum.	1	0	9
xiv. Perform bimanual examination to check size and firmness of uterus.	1	0	9
xv. Rinse the tissue with water or saline, if necessary.	1	0	9
xvi. Quickly inspect the tissue removed from the uterus to be sure the uterus is completely evacuated.	1	0	9
xvii. If no POC are seen, reassess situation to be sure it is not an ectopic pregnancy.	1	0	9
xviii. Gently insert speculum and check for bleeding.	1	0	9
xix. If uterus is still soft or bleeding persists, repeat steps 3–10.	1	0	9
**VII. POST-PROCEDURE INFECTION PREVENTION**			

i. Before removing gloves, dispose of waste materials in a leakproof container or plastic bag.	1	0	9
ii. Place all instruments in 0.5% chlorine solution for 10 minutes for decontamination.	1	0	9
iii. Decontaminate or dispose of needle or syringe:<br>If reusing needle or syringe, fill syringe (with needle attached) with 0.5% chlorine solution and submerge in solution for 10 minutes for decontamination.<br>If disposing of needle and syringe, flush needle and syringe with 0.5% chlorine solution three times, then place in a puncture-proof container.	1	0	9
iv. Sterilization of the MVA instruments (describe)	1	0	9
v. Attach used cannula to MVA syringe and flush both with 0.5% chlorine solution.	1	0	9
vi. Detach cannula from syringe and soak them in 0.5% chlorine solution for 10 minutes for decontamination.	1	0	9
vii. Empty POC into utility sink, flushable toilet, latrine or container with tight-fitting lid.	1	0	9
viii. Immerse both gloved hands in 0.5% chlorine solution. Remove gloves by turning inside out.<br>If disposing gloves, place them in a leakproof container or plastic bag.<br>If reusing surgical gloves, submerge them in 0.5% chlorine solution for decontamination.	1	0	9
ix. Wash hands thoroughly with soap and water and dry with a clean, dry cloth or air dry.	1	0	9
**VIII. POST-PROCEDURE MONITORING**			
i. Allow the patient to rest comfortably for at least 30 minutes where her recovery can be monitored.	1	0	9
ii. Check for bleeding and ensure that cramping has decreased before discharge.	1	0	9
**IX. POST-PROCEDURE COUNSELING**			
i. Instruct patient regarding post abortion care and warning signs.	1	0	9
ii. If patient desires another pregnancy instruct her to wait at least six months before attempting to become pregnant.	1	0	9
iii. Tell her when to return if followup is needed and that she can return anytime she has concerns.	1	0	9
iv. Discuss reproductive goals and, as appropriate, provide family planning.	1	0	9

Comments:

END OF CHECKLIST
